# Early Phthalates Exposure in Pregnant Women Is Associated with Alteration of Thyroid Hormones

**DOI:** 10.1371/journal.pone.0159398

**Published:** 2016-07-25

**Authors:** Po-Chin Huang, Chih-Hsin Tsai, Wei-Yen Liang, Sih-Syuan Li, Han-Bin Huang, Pao-Lin Kuo

**Affiliations:** 1 National Environmental Health Research Center, National Institute of Environmental Health Sciences, National Health Research Institutes, Miaoli, Taiwan; 2 Research Center for Environmental Medicine, Kaohsiung Medical University, Kaohsiung, Taiwan; 3 School of Public Health, National Defense Medical Center, Taipei, Taiwan; 4 Department of Obstetric and Gynecology, National Cheng Kung University Hospital and College of Medicine, Tainan, Taiwan; INIA, SPAIN

## Abstract

**Introduction:**

Previous studies revealed that phthalate exposure could alter thyroid hormones during the last trimester of pregnancy. However, thyroid hormones are crucial for fetal development during the first trimester. We aimed to clarify the effect of phthalate exposure on thyroid hormones during early pregnancy.

**Method:**

We recruited 97 pregnant women who were offered an amniocentesis during the early trimester from an obstetrics clinic in southern Taiwan from 2013 to 2014. After signing an informed consent form, we collected amniotic fluid and urine samples from pregnant women to analyze 11 metabolites, including mono-ethyl phthalate (MEP), mono-(2-ethyl-5-carboxypentyl) phthalate (MECPP), mono-(2-ethylhexyl) phthalate (MEHP), mono-butyl phthalate (MnBP), of 9 phthalates using liquid chromatography/ tandem mass spectrometry. We collected blood samples from each subject to analyze serum thyroid hormones including thyroxine (T_4_), free T_4_, and thyroid-binding globulin (TBG).

**Results:**

Three phthalate metabolites were discovered to be >80% in the urine samples of the pregnant women: MEP (88%), MnBP (81%) and MECPP (86%). Median MnBP and MECPP levels in pregnant Taiwanese women were 21.5 and 17.6 μg/g-creatinine, respectively, that decreased after the 2011 Taiwan DEHP scandal. Results of principal component analysis suggested two major sources (DEHP and other phthalates) of phthalates exposure in pregnant women. After adjusting for age, gestational age, TBG, urinary creatinine, and other phthalate metabolites, we found a significantly negative association between urinary MnBP levels and serum T_4_ (β = –5.41; p-value = 0.012; n = 97) in pregnant women using Bonferroni correction.

**Conclusion:**

We observed a potential change in the thyroid hormones of pregnant women during early pregnancy after DnBP exposure. Additional study is necessitated to clarify these associations.

## Introduction

Phthalates are used in many daily products including plastics, toys, medical equipment, personal care products, cosmetics, and food package film [1]. Reports on the endocrine-related effects of phthalate exposure on human health, especially for susceptible populations, have rapidly increased that are worthy of further investigation [2]. In 2011, a scandal involving phthalate-tainted food occurred in Taiwan; it was controlled within 3 months through actions including the restriction and regulation of six commercial phthalates [3]. Several studies have revealed that the 2011 Taiwan di-(2-ethylhexyl) phthalate (DEHP) scandal has permanently changed the profiles of phthalate exposure and potential health effects in DEHP-exposed newborns and children, as well as in the general Taiwanese population [1, 4–6], but little information is available for pregnant women.

Epidemiologic studies have revealed that phthalates may negatively alter thyroid hormones in general and susceptible populations, especially in pregnant women [4, 7–9]. Some experimental studies have further confirmed that certain phthalates, including DEHP and di-*n*-butyl phthalate (DnBP), have antithyroid activity occurring through several possible mechanisms, such as interruption of the sodium-iodide symporter and upregulation of thyroid-related genes [10–11]. Maternal thyroid hormones in early pregnancy played a critical role in early fetal development [12–14]. Some studies have further indicated that pregnant women might be exposed to different phthalate levels during different stages of pregnancy, and that this might affect fetal development during critical windows [9,15–16]. We aimed to evaluate the effects of phthalates exposure on thyroid hormones in early pregnancy and analyze phthalate exposure levels in pregnant women after the 2011 Taiwan DEHP scandal by testing urine and amniotic fluid samples.

## Materials and Methods

### Ethics Statement

The study protocol was approved by the research ethics committee of the National Health Research Institutes (No. EC1020302) and Institutional Review Board of National Cheng Kung University Hospital (No. A-ER-102-104) in Taiwan, and a written informed consent was obtained from each participant prior to study enrollment.

### Subject Recruitment

Our participants were pregnant women for whom the blood biochemical examination (alpha fetal protein and free β-hCG) results were abnormal, or whose advanced maternal age (> 35 years) suggested the need to undergo amniocentesis based on the clinical suggestion of gynecologists at the National Cheng Kung University Hospital. Pregnant women with preeclampsia and abnormal chromosome diseases (e.g., Down’s syndrome) were excluded. All the fetuses of our participants were diagnosed as healthy after examination of chromosomes in their amniotic fluid samples. All participants (N = 97) were interviewed and received explanations of the benefits and risks of participating in the Tainan Birth Cohort (TBC 2013–14) project. All the fetuses of our participants were diagnosed as healthy after we examined the chromosomes in their amniotic fluid samples.

### Questionnaire

During an interview, participants provided information regarding personal characteristics (age, education, occupational history, social economic status, etc.), pregnancy history (gestational age, time to pregnancy, menarche age, and parity), lifestyle habits (tobacco use, passive smoking, and alcohol intake), and exposure history (whether exposed to DEHP-tainted products before the DEHP episode and nutritional supplement consumption, such as a vitamin complex and folic acid) to adjust for potential confounding factors.

### Sample Collection

At the beginning of amniocentesis, gynecologists drew 2 mL of amniotic fluid into a 5-mL polypropylene (PP) syringe, and immediately transferred it into amber glass bottles to analyze the levels of phthalate metabolites. Approximately 30 min before or after performing amniocentesis, we collected 20–30 mL urine samples in disposal PP vessels and immediately transferred them into 12 mL amber glass bottles for analysis of phthalate metabolites and creatinine. To prevent contamination of the urine samples, all amber glassware was prewashed with methanol, acetonitrile, and acetone. Furthermore, we obtained 20-mL blood samples from each participant in the clinic via venipuncture that were placed into PP tubes containing no anticoagulant and allowed to settle for 40 minutes, and additional centrifugation was then performed. Serum samples were analyzed for thyroid hormone and its binding globulin. All serum and urine samples were collected from each subject at the same time and stored at −80°C and −20°C, respectively.

### Analysis of Phthalate Metabolites

We used a published method [1] to analyze the levels of 11 urinary phthalate metabolites using an isotope dilution standard and online solid phase extraction, coupled with liquid chromatography electrospray ionization tandem mass spectrometry (LC-ESI-MS/MS). Eleven phthalate metabolites including mono-benzyl phthalate (MBzP), mono-iso-butyl phthalate (MiBP), mono-*n*-butyl phthalate (MnBP), mono-ethylhexyl phthalate (MEHP), mono-(2-ethyl-5-oxo-hexyl) phthalate (MEOHP), mono-(2-ethyl-5-hydroxyhexyl) phthalate (MEHHP), mono-(2-ethyl-5-carboxypentyl) phthalate (MECPP), mono-(2-carboxymethylhexyl) phthalate (MCMHP), mono-ethyl phthalate (MEP), mono-methyl phthalate (MMP), and mono-iso-nonyl phthalate (MiNP) representing exposure to 7 commonly used phthalates (DEHP, DnBP, DiBP, BBzP, DEP, DMP, and DiNP) were measured in each urine sample. After the urine sample was thawed and sonicated for 10–15 min, the urine sample (100 μL) was loaded into a 2-mL glass vial containing ammonium acetate (AA; 20 μL, >98%; Sigma Aldrich Lab, Inc, St. Louis, MO, USA), β-glucuronidase (10 μL, *Escherichia coli* K12, Roche Biomedical, Mannheim, Germany), 11 mixed phthalate metabolite standards, and 10 mixed isotopic (^13^C_4_) phthalate metabolite standards as the internal standards (100 μL; Cambridge Isotope Lab, Inc, Andover, MA, USA). After the sample was incubated (37°C, 90 min), a 270-μL solution (5% acetonitrile [ACN], Merck, Darmstadt, Germany) with 0.1% formic acid (FA, Merck, Darmstadt, Germany) was added and sealed with a PTEF cap for analysis. We used an online system that was coupled with LC-ESI-MS/MS (Agilent 1200/API 4000, Applied Biosystems, Foster City, CA, USA). We used two columns in our online system. One C18 column (Inertsil ODS-3, 33 × 4.6 mm, 5 μm, GL Science, Tokyo, Japan) was used to extract and clean our sample, and an analytical column (Inertsil Ph, 150 × 4.6 mm, 5 μm, GL Science, Tokyo, Japan) was used to separate different phthalate metabolites. The gradient program of the clean-up column was listed as follows: 100% solution A (5% ACN + 0.1% FA) (0–7 min), 100% solution B (90% ACN + 0.1% FA) (7–9 min), 100% solution A (9–10 min) and continued to 12 min. The flow rate was set at 1000 μl/min. The analytical column gradient program was listed as follows: 100% solution C (50% ACN + 10 mmole AA) (0–3.6 min), 100% solution D (95% ACN + 10 mmole AA) (3.6–8.6 min), 100% solution C (8.6–9 min) and continued to 12 min. We used a negative multiple reaction–monitoring model for mass spectroscopy detection. The ion pair of each phthalate metabolites was listed as follows: MEHP (277/134), MEOHP (291/143), MEHHP (293/121), MECPP (307/159), MCMHP (307/113), MnBP (221/71), MiBP (221/71), MEP (193/121), MiNP (291/121), MBzP (255/183), and MMP (179/107). The detection limits of the metabolites were 0.7, 0.3, 0.3, 0.3, 0.1, 1, 1, 0.3, 0.1, 0.3, and 0.3 ng/mL, respectively. One blank, repeated quality control (QC) sample was included in each batch of analyzed samples. Concentrations of blank samples was to be less than 2 fold the method detection limit. The QC sample was spiked in pooled urine samples with a mixture of phthalate metabolite standards (20–50 ng/mL) in each sample. The relative percent difference for the repeated sample, as well as recovery of the QC sample, was to be less than ±30%.

### Serum Thyroid Hormones and Creatinine Analysis

Urinary creatinine levels and serum thyroid hormones were measured by a Taiwan Accreditation Foundation–certified laboratory (No.: 1447 & 1673), which had been recognized by the International Laboratory Accreditation Cooperation Mutual Recognition Arrangement [17]. Two milliliters of urine that had been stored at −20°C was analyzed using combined clinical chemistry and immunoassay tests (Modular Analytics Serum Work Area; Roche Diagnostics). One milliliter of serum sample was analyzed for triiodothyronine (T_3_), thyroxin (T_4_), free T_4_ (FT_4_), thyroid stimulating hormone (TSH), and thyroid binding-globulin (TBG) using an electrochemiluminescence immunoassay (ECLIA) (Elecsys 2010 and Modular Analytics E170; Roche Diagnostics). We analyzed the urinary creatinine again to confirm if an abnormal creatinine level was reported.

### Statistical Analysis

Demographic factors, exposure, and outcome variables were evaluated for normal distribution and outliers. Age, age of menarche, length of gestation, time to pregnancy, and pregnancy and delivery history were evaluated as continuous variables, while education, annual family income, smoking status, pre- and post-pregnancy alcohol consumption, residential and workplace building characteristics, vitamin and folic acid consumption, and DEHP-tainted product consumption history were evaluated as nominal variables. We calculated not detectable (ND) levels as half of the detection limit of each phthalate metabolite, and detectable rate as the number of urine samples with levels of each phthalate metabolite higher than the detection limit divided by all analyzed urine samples. We calculated sum (∑) DEHP metabolites by the amounts of all five DEHP metabolites, and ∑DBP metabolites by adding concentrations of MnBP and MiBP. All measured phthalate metabolites were logarithm (log)-transformed to approximate a normal distribution. The T_4_ and TBG concentrations were a normal distribution, while TSH, T_3_, and free T_4_ or urinary creatinine levels were log-transformed to approximate a normal distribution. Pearson correlation coefficients were used to assess the associations among age, gestation, each thyroid hormone level, and each urinary phthalate metabolite level. Principal component analysis (PCA) was applied to assess the potential sources of exposure to different phthalates. Physiological factors or variables significantly correlated with urinary phthalate metabolites or thyroid hormones were included in the multiple regression model. We included most important sources of phthalates exposure in our final model. We also evaluated the correlation between adjusted creatinine and unadjusted phthalate metabolite levels. In addition, a multiple linear regression analysis was used to adjust for significant covariates. We used both adjusted creatinine and unadjusted phthalate metabolite levels in different regression models to evaluate the influence of urinary creatinine. We used Bonferroni correction to express the two-sided significance level at P< 0.05/k (k: the number of phthalate metabolites in the regression model). Commercially available statistical software (SPSS version 22.0; SAS Institute, Cary, NC, USA) was used for statistical analysis.

## Results

### Demographic Characteristics of Participants

The participants’ mean age and age of menarche were 35.1 ± 3.5 (25.5–43.4) years and 13.1 ± 1.1 (11–16) years, respectively. Upon recruitment, the average duration of gestation was 18.3 ± 1.4 (16–24) weeks. The average number of pregnancies and childbirths per participant were 1.8 ± 0.9 and 0.7 ± 0.5, respectively. Most of our subjects were well educated (95.9% graduated from university) and were considered to have greater than a middle-class socioeconomic background (83.5%). Before pregnancy, only 2 participants were active smokers (2.1%); 16 participants reported passive smoke exposure (16.5%); and 1% drank alcohol. After pregnancy, none was an active smoker or drank alcohol, but 21 participants reported passive smoke exposure (21.6%). Approximately 16.5% had stayed in a newly decorated home 1 year before the study; 13.4% had eaten DEHP-tainted products, mainly fruit jam before the 2011 Taiwan DEHP scandal. A total of 37.1% had taken a vitamin complex or folic acid during pregnancy. None had a family or medical history of thyroid diseases (Table 1). We found no significant difference in the levels of the 11 urinary phthalate metabolites between different confounders, e.g., passive smoking, nutrition supplementation, or home or workplace characteristics. None of our participants was working at a plastics-, chemical-, or cosmetics-related job.

**Table 1:** Demographic Characteristics of Participating Pregnant Taiwanese Women (N = 97).

Characteristics	Mean ± SD/N (%)
**Continuous variables (Mean ± SD)**	
Age (y)	35.1 ± 3.5
Menarche age (y)	13.1 ± 1.1
Duration of gestation (weeks)	18.3 ± 1.4
Time to pregnancy (months)	27.8 ± 30.1
Pregnancies and births	
Number of current pregnancies	1.8 ± 0.9
Number of current births	0.7 ± 0.5
**Nominal variables [N (%)]**	
Education	
≤Junior high school	1 (1.0)
Senior high school	3 (3.1)
≥University/ College	93 (95.9)
Annual family income [USD]^a^	
≤15,600	16 (16.5)
15,600–31,250	51 (52.6)
≥31,250	30 (30.9)
Active smoker^b^	
Before pregnancy	2 (2.1)
After pregnancy	0
Passive smoker	
Before pregnancy	16 (16.5)
After pregnancy	21 (21.6)
Drank alcohol^c^	
Before pregnancy	1 (1.0)
After pregnancy	0
New decoration of residence or workplace during past 1 y^d^	
Home	
Moving to a new house	16 (16.5)
Recently decorated	19 (19.6)
Workplace	
Moving to a new workplace	2 (2.1)
Recently decorated	9 (9.3)
Have ever consumed DEHP-tainted products^e^	
Yes	13 (13.4)
No	84 (86.6)
Have ever taken a vitamin complex^f^	
Yes	39 (40.2)
No	58 (59.8)
Have ever taken folic acid^f^	
Yes	36 (37.1)
No	61 (62.9)
Family or personal medical history of thyroid disease	
Yes	0 (0)
No	97 (100)

^a^Currency exchange rate of USD to new Taiwan dollar is 1:32

^b^Active smoker was defined as someone who consumed a cigarette >1 time per day

^c^Drank alcohol was defined as someone who consumed >100 mL of alcohol per week

^d^New decoration of residence or workplace means moved into a new building

^e^Have ever consumed DEHP-tainted products means before the 2011 Taiwan DEHP scandal

^f^Have ever consumed the following nutritional supplements during the past 1 month.

### Levels of 11 Phthalate Metabolites in Urine and Amniotic Fluid Samples

**Fig 1:**
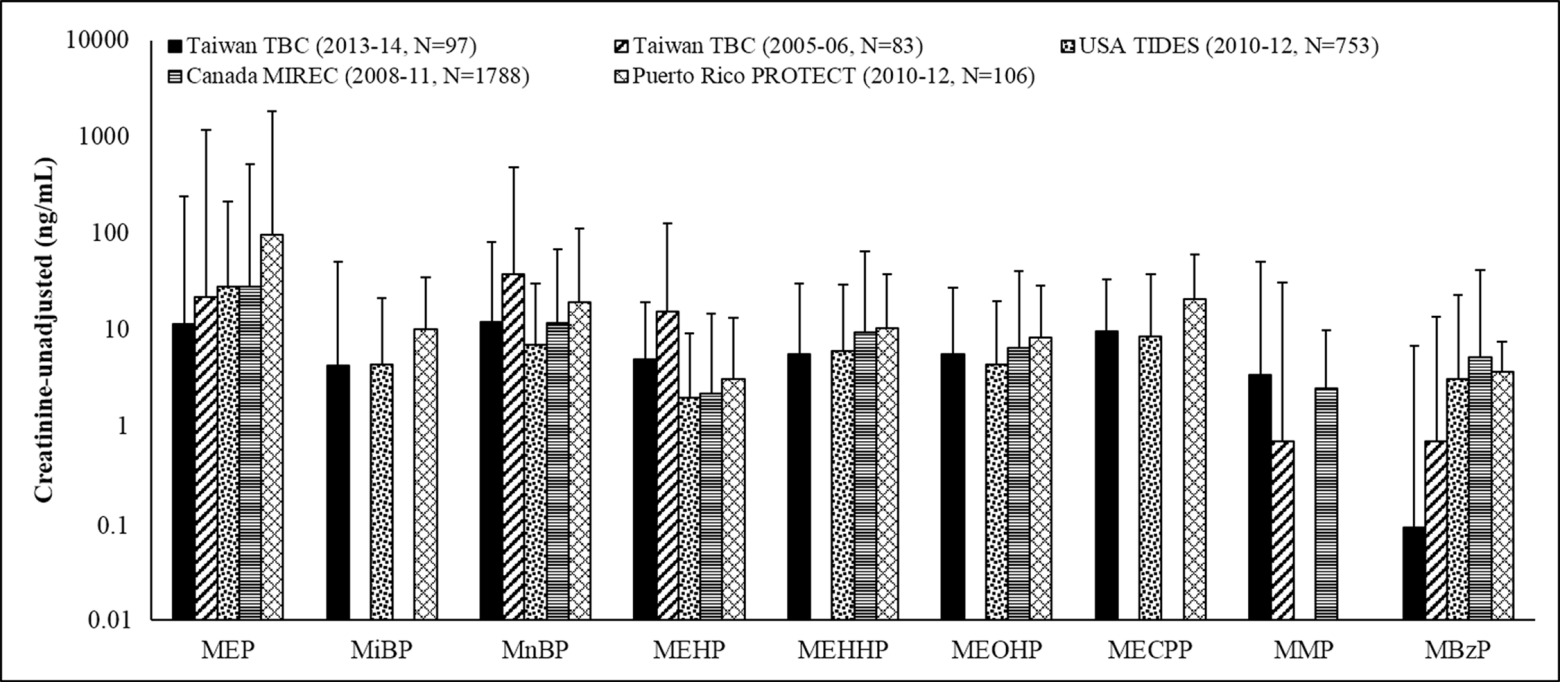
Comparison of urinary phthalate metabolites (ng/mL) at the first trimester in pregnant women in Taiwan, Unite States, Canada and Puerto Rico. TBC: Tainan Birth Cohort; TIDES: The Infant Development and the Environment Study; MIREC: Maternal-Infant Research on Environmental Chemicals; PROTECT: Puerto Rico Testsite for Exploring Contamination Threats.

The detectable rates of 11 phthalate metabolites in all urine samples ranged from 15.3% to 87.8% and were as follows: MEP (87.8%), MnBP (80.6%), MiBP (65.3%), MEHP (71.4%), MEHHP (75.5%), MEOHP (76.5%), MECPP (85.7%), MCMHP (24.5%), MMP (69.4%), MBzP (18.4%), and MiNP (15.3%) (Table 2). Distribution and median levels with and without creatinine adjustments for the 11 urinary phthalate metabolites in all participants were as follows: MnBP, 21.5 µg/g creatinine (12.3 ng/mL); MiBP, 7.4 µg/g creatinine (4.4 ng/mL); MEP, 21.0 µg/g creatinine (11.2 ng/mL); MEHP, 7.2 µg/g creatinine (5.1 ng/mL); MEHHP, 11.1 µg/g creatinine (5.7 ng/mL); MEOHP, 9.4 µg/g creatinine (5.6 ng/mL); MECPP, 17.6 µg/g creatinine (9.5 ng/mL); and MMP, 6.2 µg/g creatinine (3.4 ng/mL). However, MBzP, MCMHP, or MiNP was not detectable (ND) (Table 2). Levels of urinary MnBP and MEP were the highest of all measured metabolites, followed by MECPP, MEHHP, and MEOHP, indicating that the participants were predominantly exposed to the phthalates DnBP, DEP and DEHP. We found that levels of phthalate metabolites in our subjects increased after adjusting the creatinine levels. In addition, the median creatinine level for our participants was 50 mg/dL; however, approximately 30% of the participants had creatinine levels lower than 30 mg/dL. Because positive and high correlations were found between adjusted and unadjusted creatinine levels for all urinary phthalate metabolites (S1 Table), we used the unadjusted creatinine phthalate metabolites levels for further analysis to avoid potential bias from urinary creatinine. We found that median levels of urinary MBP (MiBP + MnBP), MEHP, MEP, and MMP in our participants (TBC 2013–14) decreased 4.8-, 5-, 1.9-, and 2-fold, respectively, compared with those in TBC 2005–06 [8], but similar levels were observed for urinary MBzP (Fig 1 and S2 Table).

**Table 2:** Distribution of creatinine-adjusted levels (μg/g creatinine, or ng/ml) of urinary phthalate metabolites in pregnant women (N = 97), and amniotic fluid phthalate metabolites (ng/ml).

Phthalate metabolites^a^	Maternal urine						Amniotic fluid			
	Detectable rate (%)	Selected percentiles					Detectable rate (%)	Selected percentiles		
		25th	50th	75th	95th	Max		75th	95th	Max
MMP	69.1	ND^b^	6.3 (3.4)	14.4 (6.8)	49.3 (51.6)	115 (75.3)	1.0	ND	ND	5.29
MEP	87.6	7.6	21.0 (11.5)	43.2 (22.7)	233 (246)	693 (686)	3.1	ND	ND	3.21
MiBP	64.9	ND	7.4 (4.3)	19.4 (12.2)	69.9 (50.8)	274 (142)	0	ND	ND	ND
MnBP	80.4	9.6 (3.2)	21.0 (12.1)	42.8 (25.6)	73.5 (82.3)	180 (102)	37.1	2.53	7.17	12.52
MBzP	18.6	ND	ND	ND	12.8 (6.8)	95.8 (51.7)	0	ND	ND	ND
MEHP	71.1	ND	7.2 (5.0)	19.8 (9.9)	42.3 (19.6)	76.6 (46.0)	49.5	9.62	24.4	29.78
MEHHP	75.3	2.2 (1.1)	10.8 (5.7)	17.7 (12.0)	33.2 (30.2)	81.3 (68.9)	0	ND	ND	ND
MEOHP	75.3	3.2 (1.2)	9.5 (5.6)	16.4 (11.4)	35.7 (27.5)	56.2 (36.4)	0	ND	ND	ND
MECPP	85.6	8.7 (4.0)	17.7 (9.7)	30.7 (20.4)	101 (33.9)	840 (143)	1.0	ND	ND	2.07
MCMHP	24.7	ND	ND	1.7 (1.2)	6.4 (5.3)	601 (102)	0	ND	ND	ND
MiNP	15.5	ND	ND	ND	27.7 (10.3)	56.5 (13.1)	5.2	ND	2.49	8.15

^a^Abbreviations: mono-methyl phthalate (MMP), mono-ethyl phthalate (MEP), mono-iso-butyl phthalate (MiBP), mono-n-butyl phthalate (MnBP), mono-benzyl phthalate (MBzP), mono-ethylhexyl phthalate (MEHP), mono-(2-ethyl-5-hydroxyhexyl) phthalate (MEHHP), mono-(2-ethyl-5-oxo-hexyl) phthalate (MEOHP), mono-(2-ethyl-5-carboxypentyl) phthalate (MECPP), mono-(2-carboxymethylhexyl) phthalate (MCMHP), mono-iso-nonyl phthalate(MiNP).

^b^Creatinine unadjusted phthalate metabolites value is presented in parentheses. Not detectable (ND) was calculated as half of detection limit (LOD).

Few phthalate metabolites were detected in amniotic fluid sample (Table 2). The detectable rates of 11 phthalate metabolites in all amniotic fluid samples ranged from 0% to 37.1% (MnBP) and 49.5% (MEHP). We found that only the MEHP and MnBP metabolites were at the 75th percentile (9.62 and 2.53 ng/mL, respectively) and were above the LOD.

### Thyroid Hormone Level

Approximately 90% of the participants’ thyroid hormone levels for T_3_, T_4_, and TSH were within reference values for the general population (Table 3). We found the FT_4_ levels during the first quartile (0.69 ng/dL) for our participants were below the lowest level for the general population, indicating that around 27% of our participants might have had a mild thyroxine insufficiency (i.e., subclinical hypothyroidism).

**Table 3:** Distribution of serum thyroid hormones and urinary creatinine in participants (N = 97) at the first trimester.

Items	Selected percentiles							Reference range^a^
	Min	5th	25th	50th	75th	95th	Max	
TSH (μIU/ml)	0.01	0.11	0.73	1.25	1.70	2.79	6.15	0.35–4.94
T_3_ (ng/dl)	43	76	106	126	144	184	259	58–159
T_4_ (μg/dl)	4.60	6.90	8.10	8.79	10.16	11.54	13.16	4.87–11.72
Free T_4_ (ng/dl)	0.52	0.57	0.69	0.77	1.01	1.11	1.17	0.7–1.48
TBG (μg/ml)	16.1	20.3	30.0	36.2	41.4	50.8	60.8	15.8–25.4
Creatinine (mg/dl)	8	13	25	50	96	168	229	60–250

Abbreviations: TSH: thyroid stimulating hormone; T_3_: triiodothyronine; T_4_:Thyroxine; Free T_4_: free thyroxine; TBG: thyroxine-binding globulin.

^a^Clinical normal range for normal adults.

### Correlation between Phthalate Metabolites and Thyroid Hormones

We found a significantly positive correlation between T_3_ and T_4_ levels (R = 0.423; p-value < 0.05), T_3_ and FT_4_ levels (R = 0.249; p-value < 0.05), T_3_ and TBG levels (R = 0.582; p-value < 0.001), and T_4_ and TBG levels (R = 0.642; p-value < 0.001) (Table 4). A marginally significant and negative correlation was found between T_4_ and urinary MnBP (R = −0.179; p-value < 0.10). Increasing age was correlated with T_3_ levels (R = 0.212; p-value < 0.05). Urinary MiBP was significantly and positively associated with FT_4_ (R = 0.313; p-value < 0.05) and age (R = 0.216; p-value < 0.05). In addition, no correlation was found between gestational age and thyroid hormone levels or urinary phthalate metabolites. Therefore, these parameters were considered continuous variables for further adjustment.

**Table 4:** Pearson correlation between thyroid hormone, age, duration of gestation, time to pregnancy and urinary phthalate metabolite levels (ng/ mL, N = 97)^a^.

Parameters^b^	Urinary phthalate metabolites											Thyroid hormone					Pregnant women		
	MMP	MEP	MiBP	MnBP	MEHP	MEHHP	MEOHP	MECPP	MBzP	MCMHP	MiNP	TSH	T_3_	T_4_	Free T_4_	TBG	Age	Gestation	Time to pregnancy
MMP	1																		
MEP	0.313*	1																	
MiBP	0.579*	0.354*	1																
MnBP	0.344*	0.284*	0.535*	1															
MEHP	0.530*	0.211*	0.583*	0.380*	1														
MEHHP	0.448*	0.298*	0.499*	0.474*	0.669*	1													
MEOHP	0.388*	0.400*	0.427*	0.448*	0.563*	0.673*	1												
MECPP	0.195	0.192	0.195	0.198	0.524*	0.551*	0.522*	1											
MBzP	**0.227**	0.319*	0.329*	0.300*	0.320*	0.136	0.385*	0.071	1										
MCMHP	0.192	0.165	0.227*	0.097	0.315*	0.138	0.273*	0.177	0.298*	1									
MiNP	-0.047	0.141	0.150	-0.142	-0.099	-0.271*	0.053	-0.234*	0.145	0.087	1								
Thyroid hormone																			
TSH	-0.088	0.043	-0.123	0.142	-0.014	0	-0.098	-0.056	0.001	0.072	-0.032	1							
T_3_	0.093	0.052	0.107	0.017	0.080	-0.042	0.029	-0.090	-0.032	-0.048	0.180	-0.006	1						
T_4_	-0.069	-0.051	0.021	-0.179#	-0.036	-0.076	0	0.020	-0.084	0.226*	0.238*	-0.065	0.423*	1					
Free T_4_	0.078	0.121	0.313*	-0.100	0.100	-0.149	-0.137	-0.090	-0.034	-0.151	0.410*	-0.042	0.249*	0.131	1				
TBG	-0.087	-0.038	0.024	-0.019	0.042	-0.026	0.001	0.045	-0.092	0.016	0.078	0.070	0.582*	0.642*	0.088	1			
Pregnant women																			
Age	0.204*	0.123	0.216*	-0.009	0.093	0.043	0.100	-0.050	-0.016	0.062	0.104	0.041	0.212*	0.078	0.104	0.166	1		
Gestation	-0.054	-0.110	0.002	-0.046	-0.070	-0.094	0	0.002	-0.087	-0.100	-0.068	-0.048	-0.004	0.143	-0.065	0.003	0.030	1	
Time to pregnancy	-0.041	0.026	0.130	-0.140	0.073	-0.020	0.025	0.025	-0.122	0.191	0.198	0.061	0.079	0.144	0.158	0.050	0.247*	0.095	1

^a^Pearson correlation coefficients: * P< 0.05; # P< 0.10.

^b^Abbreviations were shown in the footnote of Tables 2 and 3.

### Principal Component Analysis (PCA)

**Fig 2:**
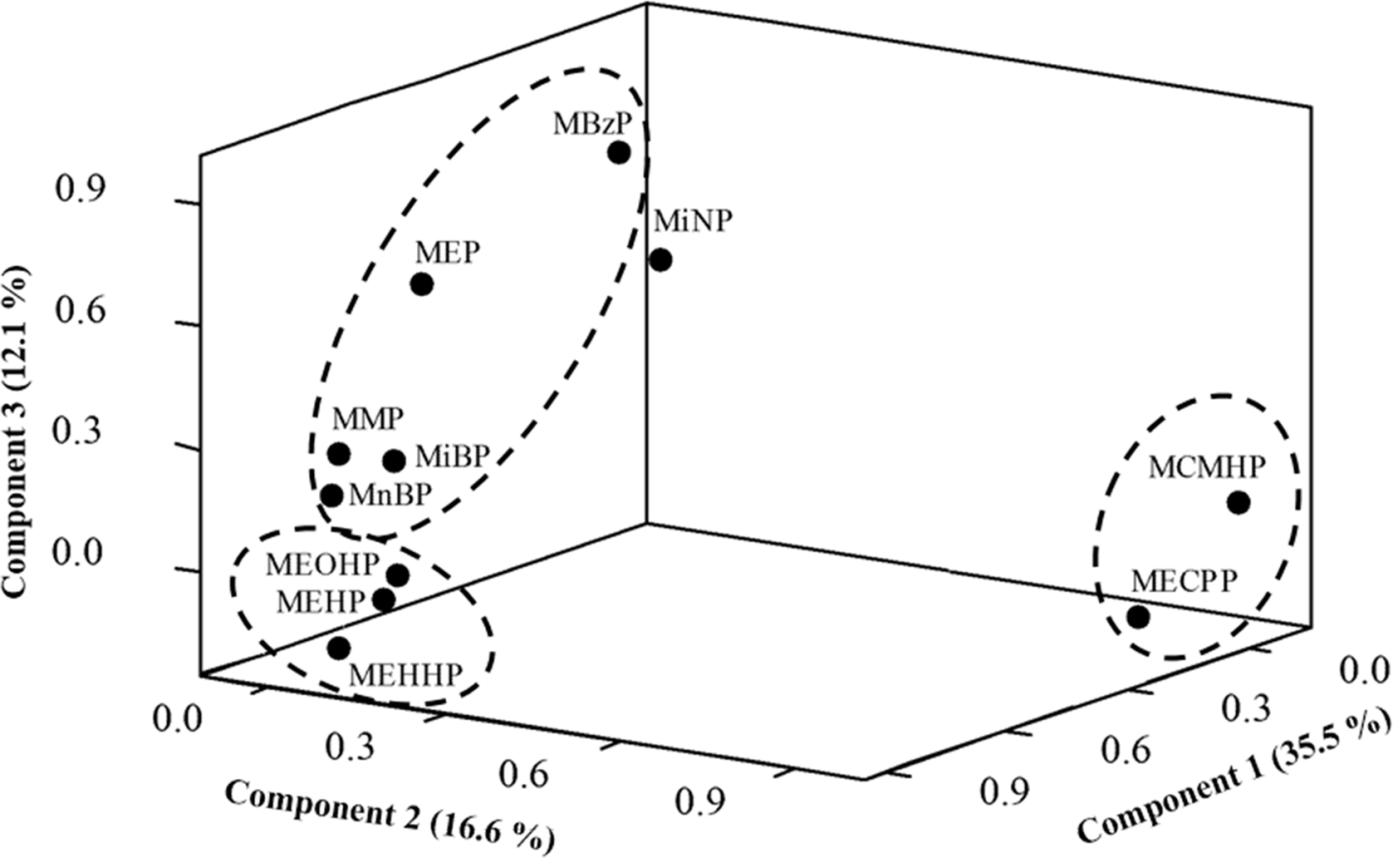
Principal component analysis of 11 urinary phthalate metabolites in 97 pregnant Taiwanese women.

Fig 2 illustrates the PCA of 11 phthalate metabolites in this study. Three principal components (PCs) accounted for 35.5% (PC1), 16.6% (PC2), and 12.1% (PC3) of the total variance. We found that PC1 was dominated by three DEHP metabolites (MEHP, MEHHP, and MEOHP); PC2 by the group of MnBP, MiBP, MEP, MMP, and MBzP; and PC3 by the group of MECPP and MCMHP. We also found profiles of major exposure to the sum DEHP metabolites, MEP and the sum of DBP metabolites (MnBP and MiBP) accounted for 32.7%, 31.5%, and 25.5%, respectively, of the total concentration of 11 phthalate metabolites in our participants (S1 Fig).

### Regression Analysis

**Fig 3:**
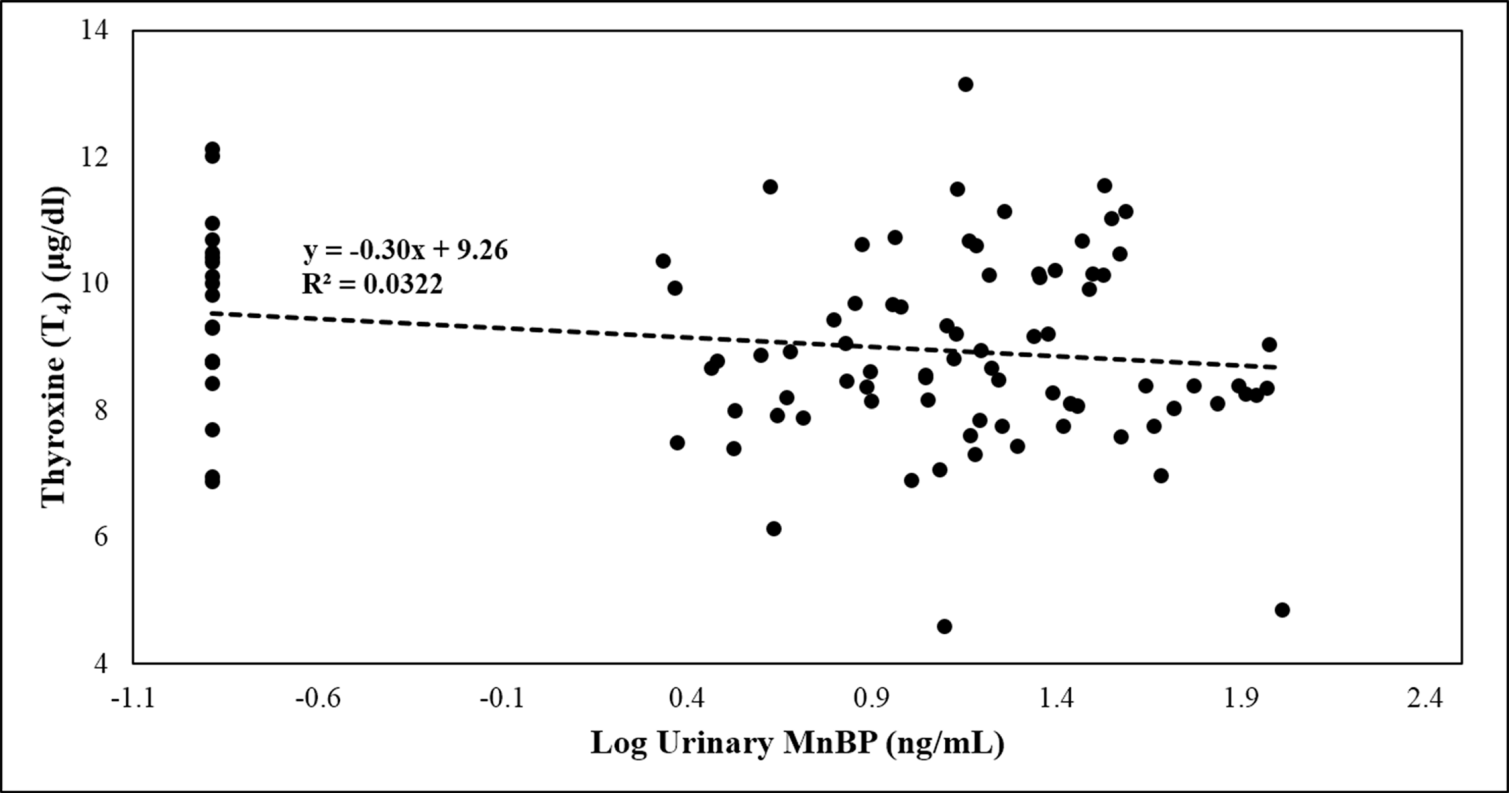
Scatter plot of correlation between serum thyroxin and urinary MnBP in pregnant women at first trimester (N = 97).

Fig 3 shows a scatter plot of serum T_4_ levels, as well as log urinary MnBP levels, with adjusted (triangle, dashed line) and unadjusted creatinine levels (circle, solid line) for each participant. To identify the major factors contributing to serum T_4_ concentrations, we used a multivariate regression model to examine the association between thyroid hormone levels and urinary phthalate metabolites (Table 5). After adjusting for age, gestational age, TBG, urinary creatinine, and three phthalate metabolites (MECPP, MEP, MiBP) in our subjects, urinary MnBP levels showed a significantly negative association with T_4_ (T_4_: β = −5.41; SE: 2.1; 95%CI: –9.59 to –1.24; p-value = 0.012; n = 97) in the participating pregnant women (Model 1). Meanwhile, a similar result was observed if using a creatinine-based phthalate metabolite level (Model 2).

**Table 5:** Multiple linear regression between serum T_4_ and corresponding urinary phthalate metabolites (N = 97; T_4_: R^2^ = 0.475).

Multiple regression	T_4_ (nmol/L)				
Model 1^a^	Beta	SE	95% CI		P-value
Intercept	-25.5	63.3	-151.2	100.2	0.688
MnBP (ng/mL)	-5.41	2.10	-9.59	-1.24	**0.012**
MECPP (ng/mL)	-0.16	2.23	-4.60	4.28	0.944
MEP (ng/mL)	0.01	2.18	-4.32	4.34	0.997
MiBP (ng/mL)	2.73	2.10	-1.44	6.90	0.197
Age	-0.37	0.45	-1.26	0.53	0.416
Gestational age	81.0	49.0	-16.3	178.3	0.102
TBG	1.45	0.18	1.10	1.81	**<0.001**
Creatinine	2.27	6.24	-10.1	14.7	0.717
Model 2^a^	Beta	SE	95% CI		P-value
Intercept	-20.1	63.7	-146.7	106.4	0.753
MnBP (μg/g-creatinine)	-5.44	2.07	-9.56	-1.33	**0.010**
MECPP (μg/g-creatinine)	-0.14	2.22	-4.55	4.27	0.949
MEP (μg/g-creatinine)	-0.02	2.16	-4.31	4.28	0.994
MiBP (μg/g-creatinine)	2.69	2.07	-1.41	6.80	0.195
Age	-0.37	0.45	-1.26	0.52	0.412
Gestational age	80.6	48.6	-16.0	177.2	0.101
TBG	1.45	0.18	1.10	1.80	**<0.001**

^a^MnBP, MECPP, MEP, and MiBP levels; urinary creatinine; and gestational age were log_10_ transformed.

## Discussion

We observed 5-fold decreases in the levels of urinary MBP and MEHP in pregnant Taiwanese women (2013–14) after the 2011 Taiwan DEHP scandal in comparison with those reported in 2005–06 [8]. We found a significantly negative association between urinary MnBP and serum T_4_ in the pregnant women during the first trimester after adjusting for age, TBG, gestational age, and exposure to other phthalates. Our data suggest that DnBP exposure may alter serum T_4_ levels during early pregnancy.

Four studies previously conducted in Taiwan (2005–2006)[8], the United States (2010–2012)[18], Canada (2008–2011) [19], and Puerto Rico (2010–2012)[20] provided exposure profiles for some phthalates in early pregnancy (Fig 1 & S2 Table). Our data showed that urinary MBP (MnBP + MiBP) and MEHP levels in our participants (Taiwan 2013–14) were nearly 5-fold lower than those reported in a previous study conducted in Taiwan from 2005 to 2006 [8], before the 2011 Taiwan DEHP scandal. Although DBP and DEHP exposure levels may vary by person, be correlated with lifestyle, or gradually decrease [21], the significant drop in metabolites of DEHP and DBP exposure in pregnant Taiwanese women might be related to the regulation and restriction of DEHP and DBP usage in certain products after the 2011 Taiwan DEHP scandal that may decrease overall phthalate exposure in the general Taiwanese population [1].

Additionally, the exposure levels of MnBP and MEHP in our participants were 1.8- and 2.6-fold, respectively, higher than those reported in pregnant American women during the first trimester [18], while those of MEP and MBzP were lower. A similar phenomenon was found for MnBP, MEHP, MEP, and MBzP, if comparing our data to a Canadian study, MIREC [19]. Exposure levels for most phthalate metabolites in pregnant Puerto Rican women in the PROTECT study [20] were higher than those reported in Taiwan, the US, and Canada, especially for urinary MEP, MnBP, and MECPP levels. However, the exposure profiles of six urinary phthalate metabolites in our participants were similar to those reported in China [22]. Some studies [23–25] have revealed that exposure to urinary MEP, MBP, and MBzP may be related to personal care products (e.g., sunscreen and lotion) and cosmetics (e.g., perfumes, nail polish, and makeup) in pregnant American women and women globally. Because BBzP is most commonly used in building materials [2], a preference for using marble and wood as flooring in Chinese societies may explain why low levels of MBzP were observed in a Chinese population [22]. Thus, the lower levels of MEP and MBzP exposure in pregnant Taiwanese women may reflect a less intense use of personal care products or cosmetics and less frequent usage of PVC in Taiwanese building materials.

We found a strong correlation for four DEHP metabolites, MEHP, MEHHP, MEOHP and MECPP, but a relative weak correlation for MCMHP. One possible explanation is the low detection rate of urinary MCMHP in our participants (approximately 25%), which is 2.56-fold lower than that of the general female Taiwanese (approximately 64%) [1]. However, all other phthalate metabolites appeared at a detection rate in our pregnant participants that was comparable to that of the general female population. Because MCMHP is a secondary metabolites of DEHP, physiological changes in pregnant women during early pregnancy may alter the metabolic ability of oxidizing DEHP. In addition, we found three principal components that explained 64.2% of the total variance. DEHP being the predominant phthalates in food products [1], food may be the major contributor to PC1 in pregnant Taiwanese women. PC2 consists of complex phthalate metabolites, including MnBP, MiBP, MEP, MMP, and MBzP, and consumer products may contain different phthalates [26]. Personal care products, solvents, and medicine coatings DnBP, DiBP, and DEP [1]; polyvinyl chloride flooring contains BBzP[1]; and milk products, snacks, and noodles contain DMP [27]. This indicates that Taiwanese women in the early stages of pregnancy are exposed to a wide range of phthalates from daily consumer products.

Our data are consistent with several studies on phthalate metabolites in human amniotic fluid samples [28–29]. Three possible reasons exist for the decrease in correlations in the current study. One is that the difference in the ability of fetuses to metabolize phthalates may vary by species. A strong correlation between MEHP and MnBP in maternal urine and amniotic fluid samples were reported in rats [30–31]. However, some studies demonstrated that different species of mammals can have different placental barriers, such as hemoendothelial and endothelial barriers, leading to dissimilar levels of environmental pollutants in amniotic fluid [32–35].Rodents are the most frequently used models for placenta research because they possess hemochorial placentas, but these are different from the human placenta and therefore the applicability of these studies is often overvalued [35]. Another study used a human placenta perfusion system to measure the distribution of four phthalate monomers (MEHP, MnBP, MEP, and MMP) between the umbilical cord plasma and placental tissue, and suggested that this distribution depended on their physicochemical properties [36]. A second possible explanation is that the fetal liver might not be highly developed in early pregnancy. In most studies, the amniotic fluid samples contained a low levels of the oxidative metabolites of DEHP. The low detectable rate of the secondary metabolites of DEHP and DiNP reflects the possibility that the development of fetal liver detoxification, like glucuronidation, might be incomplete in early pregnancy [28, 30–31, 37]. Third, one study revealed potential contamination of urinary MEHP in the laboratory [31], meaning that correlations may have been overestimated. Nevertheless, all previous studies have indicated that DEHP and DBP can penetrate placenta and cause uterine exposure in early pregnancy.

We also observed the proportion of subclinical hypothyroidism in our subjects was nearly 2-fold less than (27% versus >50%, Table 3) that reported in a previous study [8]. We found a significantly negative association between urinary MnBP and serum T_4_ in pregnant women during the first trimester after adjusting for age, TBG, gestational age, and other phthalate exposures. Some studies have reported a negative association between DEHP or DnBP and thyroid hormones (such as T_4_ and T_3_) in the general Danish and American population, and in American pregnant women [7,9,38]. One longitudinal study indicated that significant inverse associations were observed between FT_4_ and DEHP metabolites during the third trimester [9]. The influence of a low-dose phthalate exposure on thyroid hormones was consistent with epidemiologic studies that indicated the thyroid gland could be affected by multiple phthalate exposures. About 9.3% of TSH level in our subject was above 2.5 μIU/ml, which is a new guideline of subclinical hypothyroidism in the infertile female [39]. Subclinical hypothyroidism or phthalates exposure may increase the risk of miscarriage in early pregnancy [22, 40–43]. As thyroid hormone is crucial to fetal neurodevelopment or essential for pregnant women during early pregnancy [12, 44–45], a control strategy for reducing phthalate exposure in pregnant women should be developed, as has been reported for children [46].

Some toxicological data provided supporting evidence on phthalate exposure, especially DnBP and DEHP, along with their effects on thyroid hormones, in pregnant animals or in vitro [10, 47–48]. Male Wistar rats exposed to high DEHP doses had significantly decreased serum T_4_ concentrations after 21 days [49], indicating thyroid hyperactivity. In vitro studies have revealed that DnBP might have possible T_3_-antagonist activity based on a thyroid hormone assay [50] and demonstrated that DnBP appeared to down-regulate human sodium/ iodide symporter (NIS) [11] and MnBP could induce the expression of thyroid hormone receptor-beta [10, 47]. Recent study revealed that DEHP disrupts the growth/ development of placenta, inhibits the proliferation of placenta and induces the apoptosis of placenta via activated MAPK in CD-1 mice [51]. As for mechanisms, some evidence has indicated that phthalate exposure might alter thyroid hormones by interacting with thyroid receptors and modulate the transcriptional activity of NIS. However, mechanism of how phthalates alter implement of zygote during early pregnancy is still unclear.

We evaluated some confounding factors such as TBG, urinary creatinine, gestational age, age, and other phthalate exposures. We found that thyroid-binding globins (TBG) were significantly and positively (β: 1.45; p-value < 0.001) associated with serum T_4_ in both models. Although TBG levels may vary or exceed the normal range during pregnancy, we did not find a significant correlation between TBG and gestational age in our participants (Table 3). Shen et al. showed MnBP could induce the expression of thyroid hormone receptor-beta [47], and Liu et al. revealed that DEHP could reduce thyroid hormones by interacting with thyroid-binding proteins [10]. It is possible that DnBP may alter human thyroxine by interacting with its receptors or TBG. Further, the serum creatinine of pregnant women may decrease by 10% and urine creatinine may vary in the first trimester because of physiological change in the glomerular filtration rate and renal blood flow [13, 52]. We used adjusted and unadjusted creatinine levels to evaluate the influence of creatinine in our model. We did not observe a significant change (<1%) in the prediction model that was consistent with a prior study [53]. A median to high correlation coefficient was observed for phthalate metabolite concentrations between adjusted and unadjusted creatinine levels that ranged from 0.84 to 0.92 (S1 Table). The gestational age of our subjects might have narrowed in a certain period, which minimized the effects of unusually diluted or concentrated urinary creatinine levels. We did not find any significant effects regarding age, gestational age, or other phthalate exposure (MEP, MiBP, and MECPP) in our prediction model on T_4_.

Our study explored the association between phthalate exposure and thyroid hormones in pregnant women 2 years after the 2011 Taiwan DEHP scandal. Although maternal thyroid hormone changes are linked to phthalate metabolite levels in urine, we still found a negative association between DnBP exposure and T_4_ during early pregnancy. The fetal thyroid is essential during all aspects of fetus development and depends entirely on maternal thyroid hormones during early pregnancy [12, 44, 54]. It is important to diminish phthalate exposure for women prepare to pregnant and pregnant women during pregnancy, especially for personal care products, cosmetic and consumer products [20, 22, 55–57].

The present study had some strengths. We included TBG as a covariance to evaluate the potential influence of the natural physiology of thyroid hormones. Second, we measured 11 urinary phthalate metabolites in pregnant women to provide a solid internal-dose of phthalate exposure and to assess aggregate phthalate exposure from various sources [23]. Third, creatinine is influenced by muscle mass, racial differences, pregnancy, and dietary meat intake [57]. We conducted a sensitivity analysis for evaluating the effects of urinary creatinine on a multivariate regression model. The MBP level in a previous study [8] was the combination of MnBP and MiBP, so an assessment of the influence of individual DnBP and DiBP metabolites could not be performed. Therefore, we analyzed MnBP and MiBP to evaluate their individual effects on thyroid function, but this analysis might decrease the correlation as well.

There were some limitations in the present study. First, our study population was hospital based and relatively small. Second, we collected only 1 serum sample and 1-spot urine sample to measure the thyroid hormone and phthalate metabolite levels. Variations in our serum and urine samples might have underestimated the observed correlation [58]. Third, although phthalate metabolite levels might vary within a few days, it has been suggested that a single urine sample is a moderate indicator for phthalate metabolite measurement in pregnant women [9, 20]. Temporal variability in phthalate metabolites might have also reduced the correlation between thyroid hormone and urinary phthalate metabolite levels.

## Conclusions

We found that the T_4_ levels in pregnant women during early pregnancy were significantly and negatively associated with urinary MnBP levels after adjusting for age, gestation age, TBG, urinary creatinine, and exposures to other phthalate. TBG has been suggested to be a confounding factor in evaluating thyroid hormones. Changes in T_4_ levels during early pregnancy might be a potential threat to fetal development or pregnant loss, and a strategy to reduce the exposure of pregnant women to phthalates should be emphasized.

## Supporting Information

S1 Fig Exposure profiles of 11 urinary phthalate metabolites in pregnant Taiwanese women (N = 97). (DOCX)

S1 Table Pearson correlation coefficients between levels of creatinine unadjusted and creatinine adjusted phthalate metabolites. (DOCX)

S2 Table Comparison of urinary phthalate metabolites (ng/mL) in pregnant women of Taiwan and other countries. (DOCX)
