# Supplementary figures and images for: Early Phthalates Exposure in Pregnant Women Is Associated with Alteration of Thyroid Hormones
Source: PLoS One. 2016 Jul 25;11(7):e0159398. doi: 10.1371/journal.pone.0159398 (PMC4959782; doi:10.1371/journal.pone.0159398)

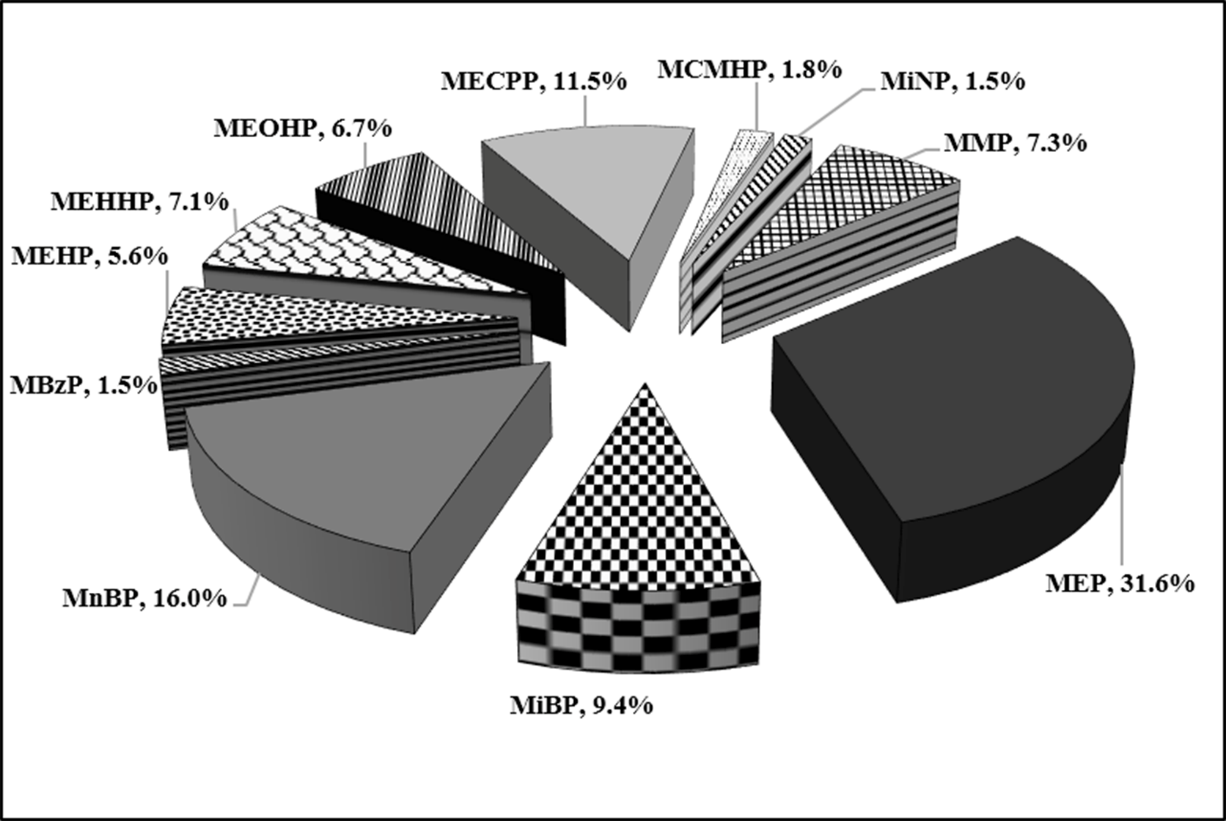


S1 Fig. Exposure profiles of 11 urinary phthalate metabolites in pregnant Taiwanese women (n=97).

Supplement: S1 Fig — (DOCX) [file pone.0159398.s001.docx]
