# Supplementary material for: Early Phthalates Exposure in Pregnant Women Is Associated with Alteration of Thyroid Hormones
Source: PLoS One. 2016 Jul 25;11(7):e0159398. doi: 10.1371/journal.pone.0159398 (PMC4959782; doi:10.1371/journal.pone.0159398)
Supplement: S1 Table — (DOCX) [file pone.0159398.s002.docx]

S1 Table. Pearson correlation coefficients between levels of creatinine unadjusted and creatinine adjusted phthalate metabolites.

| *N*=97 | MMP | MEP | MiBP | MnBP | MBzP | MEHP | MEHHP | MEOHP | MECPP | MCMHP | MiNP |
| --- | --- | --- | --- | --- | --- | --- | --- | --- | --- | --- | --- |
| MMP-c | **0.924*** | 0.134 | 0.415 | 0.152 | 0.098 | 0.415 | 0.252 | 0.131 | 0.035 | 0.067 | -0.058 |
| MEP-c | 0.107 | **0.895*** | 0.124 | 0.048 | 0.178 | 0.024 | 0.043 | 0.098 | 0.002 | 0.007 | 0.156 |
| MiBP-c | 0.460 | 0.191 | **0.926*** | 0.380 | 0.229 | 0.490 | 0.320 | 0.183 | 0.035 | 0.116 | 0.179 |
| MnBP-c | 0.171 | 0.096 | 0.360 | **0.918*** | 0.183 | 0.240 | 0.278 | 0.192 | 0.031 | -0.048 | -0.169 |
| MBzP-c | -0.030 | 0.060 | 0.048 | 0.024 | **0.862*** | 0.106 | -0.175 | 0.023 | -0.157 | 0.124 | 0.146 |
| MEHP-c | 0.340 | -0.019 | 0.362 | 0.144 | 0.175 | **0.893*** | 0.447 | 0.270 | 0.368 | 0.179 | -0.112 |
| MEHHP-c | 0.302 | 0.118 | 0.330 | 0.307 | -0.009 | 0.597 | **0.923*** | 0.473 | 0.460 | 0.002 | -0.330 |
| MEOHP-c | 0.185 | 0.193 | 0.192 | 0.230 | 0.273 | 0.467 | 0.510 | **0.873*** | 0.415 | 0.131 | 0.066 |
| MECPP-c | -0.054 | -0.065 | -0.092 | -0.079 | -0.120 | 0.344 | 0.289 | 0.192 | **0.872*** | 0.006 | -0.264 |
| MCMHP-c | -0.088 | -0.129 | -0.084 | -0.214 | 0.095 | 0.081 | -0.196 | -0.125 | -0.063 | **0.839*** | 0.082 |
| MiNP-c | -0.400 | -0.285 | -0.314 | -0.502 | -0.175 | -0.387 | -0.620 | -0.488 | -0.476 | -0.203 | **0.672*** |
