# Supplementary material for: Early Phthalates Exposure in Pregnant Women Is Associated with Alteration of Thyroid Hormones
Source: PLoS One. 2016 Jul 25;11(7):e0159398. doi: 10.1371/journal.pone.0159398 (PMC4959782; doi:10.1371/journal.pone.0159398)
Supplement: S2 Table — (DOCX) [file pone.0159398.s003.docx]

S2 Table. Comparison of urinary phthalate metabolites (ng/mL) in pregnant women of Taiwan and other countries.

| County | Age (years) | *N* | MiBP | MnBP | MEHP | MEHHP | MEOHP | MECPP | MCMHP | MEP | MBzP | MMP | MiNP |
| --- | --- | --- | --- | --- | --- | --- | --- | --- | --- | --- | --- | --- | --- |
| This study | 26-43 | 97 | 4.3 (ND-142) | 12.1  (ND-102) | 5.0  (ND-46) | 5.7 (ND-68.9) | 5.6 (ND-36.4) | 9.7 (ND-143) | ND  (ND-102) | 11.5 (ND-686) | ND  (ND-51.7) | 3.4 (ND-75.3) | ND  (ND-13.1) |
| Southern Taiwan ^a^ | 26-43 | 83 | - | 38.4 (7.3-487) | 15.5 (3.6-129) | - | - | - | - | 22.1 (ND-1195) | ND (ND-13.6) | ND (ND-31.3) | - |
| Taiwan ^b^ | 18-40 | 40 | 8.9 (ND-142) | 21.1 (ND-123) | 8.3 (ND-139) | 21.4 (ND-488) | 12.0 (ND-290) | 24.7 (ND-975) | 5.0 (ND-171) | 21.7 (ND-1258) | ND (ND-8.1) | 32.2 (ND-526) | ND (ND-5.2) |
| USA ^c^ | >18 | 753 | 4.4 | 7.0 | 2.0 | 6.1 | 4.4 | 8.6 | - | 28.4 | 3.1 | - | - |
| Canada ^d^ | 18-35 | 1788 | - | 12.0 (69.7-3100) | 2.2 (15.0-340) | 9.4 (65.7-1200) | 6.5 (41-980) | - | - | 28.0 (530-13000) | 5.2 (41.7-420) | 2.5 (10.0-1000) | ND (ND-9.2) |
| Puerto Rico ^e^ | 18-40 | 106 | 10.3 (6.0-157) | 19.3 (10.8-278) | 3.1 (1.6-50.9) | 10.5 (6.1-290) | 8.3 (5.6-259) | 20.8 (1.0-82.1) | - | 97.7 (24.2-6910) | 3.7 (1.7-59.0) | - | - |
| Japan ^f^ | 31.9 ± 4.5 | 149 | - | 48.1 (2.9-504) | 4.4 (ND-70.3) | 8.61 (0.4-89.7) | 9.2 (0.7-132) | - | - | 6.0 (0.3-1067) | 3.5 (ND-992) | 6.5 (0.4-464) | ND (ND-23.1) |
| Peru ^g^ | 14-46 | 79 | 1.2 (1.0-1.4) | - | 1.6 (1.4-1.8) | 4.1 (3.4-4.9) | 3.1 (2.6-3.7) | 10.5 (9.1-12.2) | - | 32.2 (24.9-41.6) | 1.1 (0.9-1.4) | - | - |
| Frence ^h^ | - | 279 | 53.7 (5.5-933) | 35.7 (1.5-912) | 16.7 (1.1-768) | 41.9 (1.1-1598) | 28.5 (1.5-925) | 427 (3.0-2004) | 12.3 (1.0-1212) | 43.5 (6.3-1286) | 10.1 (1.3-1178) | - | ND |

Unit: ng/ml; ^a^ Median (range); ^b^ Geometric mean (95%CI)

^a^ Southern Taiwan (sampling year: 2005-06; gestation: 27.9±2.3; trimester: 1^st^; Huang et al., 2009)

^b^ Taiwan (sampling year: 2013; General population; Huang et al., 2015);

^c^ USA (sampling year: 2010-12; gestation: <13; trimester= 1^st^; Swan et al., 2015);

^d^ Canada (median (95^th^-max); sampling year: 2008-11; gestation: 6.1-14.9; trimester= 1^st^; Arbuckle et al., 2014);

^e^ Puerto Rico (median (25^th^-max); sampling year: 2010-12; gestation: 16-20; trimester= 1^st^; Johns et al., 2015);

^f^ Japan (sampling year: 2005-08; gestation: 9-40; trimester= 1^st^-3^rd^; Suzuki et al., 2010);

^g^ Peru (GM (95% CI); sampling year: 2004; trimester= 1^st^-3^rd^; Irvin Ann et al., 2010);

^h^ French (sampling year: 2007; trimester= 1^st^-3^rd^; Zeman et al., 2013).
